# Clearance of technetium-99m-DTPA and HRCT findings in the evaluation of patients with Idiopathic Pulmonary Fibrosis

**DOI:** 10.1186/1471-2466-6-4

**Published:** 2006-02-16

**Authors:** Katerina M Antoniou, Katerina Malagari, Nikolaos Tzanakis, Kostas Perisinakis, Emmanouil K Symvoulakis, Nikolaos Karkavitsas, Nikolaos M Siafakas, Demosthenes Bouros

**Affiliations:** 1 Department of Thoracic Medicine, University Hospital of Heraklion Crete, Greece; 2 Department of Radiology, Medical School University of Athens, Greece; 3 Department of Nuclear Medicine, Medical School University of Crete, Greece; 4 Department of Pneumonology, Democritus University of Thrace, Alexandroupolis, Greece

## Abstract

**Background:**

Clearance of inhaled technetium-labeled diethylenetriamine pentaacetate (^99m^Tc-DTPA) is a marker of epithelial damage and an index of lung epithelial permeability. The aim of this study was to investigate the role of ^99m^Tc-DTPA scan in patients with Idiopathic Pulmonary Fibrosis (IPF). Our hypothesis is that the rate of pulmonary ^99m^Tc-DTPA clearance could be associated with extent of High Resolution Computed Tomography (HRCT) abnormalities, cell differential of bronchoalveolar lavage fluid (BALF) and pulmonary function tests (PFTs) in patients with IPF.

**Methods:**

We studied prospectively 18 patients (14 male, 4 female) of median age 67yr (range 55–81) with histologically proven IPF. HRCT scoring included the mean values of extent of disease. Mean values of these percentages represented the Total Interstitial Disease Score (TID). DTPA clearance was analyzed according to a dynamic study using a Venticis II radioaerosol delivery system.

**Results:**

The mean (SD) TID score was 36 ± 12%, 3 patients had mild, 11 moderate and 4 severe TID. Abnormal DTPA clearance half-time (*t*_1/2_<40 min) was found in 17/18 (94.5%) [mean (SD) 29.1 ± 8.6 min]. TID was weakly correlated with the DTPA clearance (r = -0.47, p = 0.048) and with % eosinophils (r = 0.475, p = 0.05). No correlation was found between TID score or DTPA and PFTs in IPF patients.

**Conclusion:**

Our data suggest that ^99m^Tc-DTPA lung scan is not well associated with HRCT abnormalities, PFTs, and BALF cellularity in patients with IPF. Further studies in large scale of patients are needed to define the role of this technique in pulmonary fibrosis.

## Background

Clearance of inhaled technetium-labeled diethylenetriamine pentaacetate (^99m^Tc-DTPA) is an index of lung epithelial permeability [1,2]. The technique is non invasive, relatively inexpensive with a low radiation, and easy to perform using standard equipment in clinical nuclear medicine department [3].

Abnormal ^99m^Tc-DTPA has been found in several interstitial lung diseases [4,5]. It has been used as a marker of alveolar inflammation in sarcoidosis [4] and could potentially be used as a predictor of disease progression in idiopathic pulmonary fibrosis (IPF) [5]. In interstitial lung disease (ILD), active inflammation in the lower respiratory tract results in lung injury and fibrosis. On the contrary, recent data suggests that inflammation does not play a pivotal role in IPF [6]. Evidence suggests that epithelial injury, even in the absence of inflammation is sufficient to stimulate the development of fibrosis [6].

High-resolution computed tomography (HRCT) has diagnostic and prognostic value, and should be a part of the initial evaluation of ILD [7]. The extent of IPF on HRCT correlates roughly with severity of functional impairment (i.e, FVC and DLCO [8].

The objective of this study was to explore the role of ^99m^Tc-DTPA scanning in a well-defined population of IPF patients. In addition, our aim was to determine whether DTPA clearance is correlated with HRCT score, BALF differential cell counts and pulmonary function tests (PFTs).

## Methods

### Patients

Eighteen consecutive IPF patients who were investigated at our departments were enrolled in the study. The diagnosis of IPF was based on recently established criteria [9] and confirmed by lung biopsy with video-assisted thoracoscopic surgery (VATS). The histologic appearance was that of usual interstitial pneumonia (UIP) and all patients were newly diagnosed.

All patients (14 male, 4 female) of median age 67 yr (range, 55–81), were studied prospectively. Patients were excluded from the study if they were current smokers (cessation of smoking at least 6 months prior to the measurement of DTPA clearance was a prerequisite for the enrollment).

### Spirometry

Spirometry was performed with a computerized system (MasterLab, Jaeger 2.12, Germany). The measurement was performed using standard protocols according to American Thoracic Society (ATS) guidelines [10]. Subjects did not use short acting bronchodilators within 12 hours prior to the measurements. The best of three consecutive measurements was chosen.

### HRCT in IPF patients

The HRCT slices were evaluated at five predetermined levels: the aortic arch, the tracheal carina, the pulmonary hilar, the pulmonary venous confluence, and 1–2 cm above the right hemidiaphragm. The scans were performed with 1 mm thickness and 1 to 2 sec scanning time during breath holding at end inspiration. High spatial frequency algorithm was used and images were read at window levels appropriate for pulmonary parenchyma (level-600 to-700 Hounsfield units; window 1500–1600).

HRCT scoring in IPF patients included extent of disease at each of the above mentioned levels at the level of 5% using a visual method of assessment similar to that used previously by the same [11] and other investigators [8,12-15]. Two experienced readers, first independently without any knowledge of the clinical and functional findings and subsequently- in cases in which there were discrepancies- with consensus, examined the HRCT scans.

HRCT scoring included extent of overall involvement of lung parenchyma at each HRCT slice at the nearest 5% (range 0–100) using a visual method of assessment similar to that used previously by other investigators [8,11-15]. The total interstitial disease score (TIDs) was calculated by the sum of all the above mentioned percentages divided by five-the number that represented the number of the slices evaluated. Interestingly, scores from the five HRCT slices were summed and divided by the total number of slices to calculate the average extent score for each of the variables. Similar methods of scoring have been used by Staples and coworkers [12], Lee and coworkers [13] and Wells and co-workers [8,15]. Lung parenchyma involvement was classified as 0–20%: mild (grade 1), 21–40%: moderate (grade 2), >40%: severe (grade 3). The classification of severity of disease in mild, moderate and severe is somehow arbitrarily made based on the TID obtained from the above calculations.

### ^99m^Tc-DTPA scan

An aerosol of Tc-99m diethylenetriamine pentaacetate (DTPA) was produced using a Venticis II radioaerosol delivery system (CIS bio international, Cedex, France). The radioaerosol was administered to patients for 4 minutes. A dynamic study consisting of 30 one-minute frames were then acquired by a GE Millennium MPS γ-camera (GE, Milwuakee, Wisconsin, USA).

Regions of interest (ROI) were identified on either lung and a background ROI was set. Background subtraction was employed and an exponential fit of the clearance of each lung was derived starting from the frame with peak activity. The half-time of clearance was estimated in minutes for each lung and the average value was obtained. A clearance half-time of <40 min was regarded as abnormal. A clearance half-time of less than 20 min (50% of normal) was categorized as very rapid [4,5,16].

### Bronchoalveolar lavage

Fiberoptic bronchoscopy with BAL was performed as part of routine clinical management, according to recommended guidelines and previous reports [17].

Subjects were premedicated with atropine 0.5 mg IM, and the airways were anesthetized by inhalation of 4% xylocaine. Supplemental oxygen was administered during and immediately after the procedure. Oxygen saturation and ECG tracings were monitored during the procedure. After the administration of 2% topical xylocaine, the bronchoscope was inserted transnasally and, after inspection of segmental orifices, it was wedged according to a standard protocol in the middle lobe or the lingula. Aliquots of 60 ml of sterile normal saline previously warmed to 37°C instilled through the bronchoscope, and the fluid was retrieved by mechanical suction. The standard introduction volume was 180 ml and the recovery volume >60% in all cases. The cells were recovered by gentle aspiration.

Slide preparations for differential percentage counting of cells were made in a cytocentrifuge (Aero spray, Wescor, USA) using 50-μL aliquots of the lavage cell suspensions. Two slides were stained with May-Grunewald-Giemsa stain. Differential counts were made by two independed observers for a minimum count of 500 nucleated non squamous cells. Visually identified squamous epithelial cells were not included in the total cell count. Additionally, 300 μL of BALF were processed for flow cytometric analysis.

All the above investigations (spirometry, bronchoalveolar lavage fluid, HRCT and DTPA scan) are incorporated in the routine follow-up of our hospital. An ethical approval was not necessary to be asked.

### Statistical analysis

All analyses were performed using the statistical software Stats-Disect for Windows version 1.8.9 (Camcode, Cambridge, UK). Results are expressed as mean + 1SD, or median (range), unless other indicated. The Kolmogorov-Smirnof test for normality was applied to examine data distribution. Differences between the two groups of subjects were tested using the Mann-Whitney U test. Correlation between cell and physiological variables were analyzed using Spearman's rank correlation coefficient. A p value of < 0.05 was considered as statistically significant.

## Results

The demographic and clinical characteristics of the IPF patients are shown in table 1.

The mean (SD) TID score was 36 ± 12%, 3 patients had mild, 11 moderate and 4 severe TID. Abnormal DTPA clearance half-time (*t*_1/2_<40 min) was found in 17 of the18 (94.5%) IPF patients [mean (SD) 29.1 ± 8.6 min].

**Figure 1:**
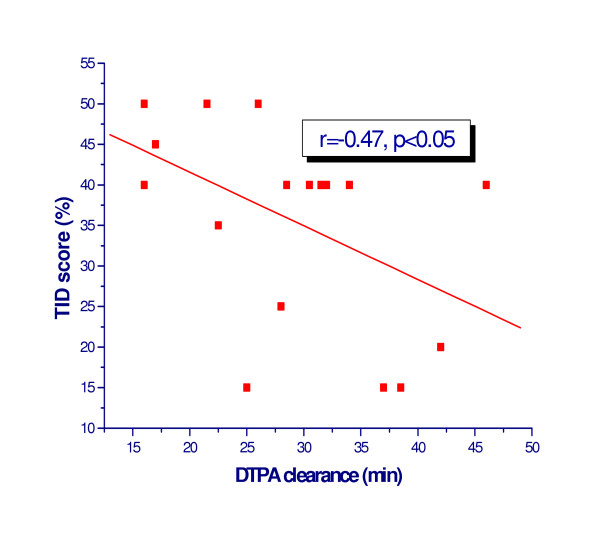
Correlation of DTPA clearance and HRCT TID score in IPF patients.

A weak association was found between TID score and DTPA clearance (r = -0.47, p = 0.048) (Figure 1). No correlation was found between TID score or DTPA and PFTs in IPF patients.

**Figure 2:**
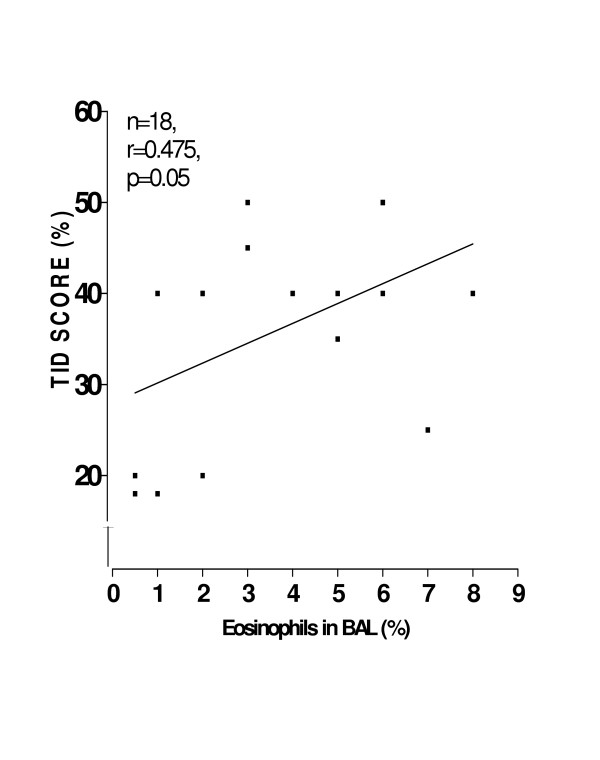
Correlation of HRCT TID score and % of eosinophils in BALF.

The total and the differential cell counts of BALF are shown in Table 2. TID score was weakly correlated with eosinophils (r = 0.475, p = 0.05) (Figure 2) and negatively with macrophages (r = -0.507, p = 0.03).

## Discussion

This is the first study to systematically evaluate newly diagnosed IPF patients with biopsy proven histology of usual interstitial pneumonia (UIP), excluding the other clinical-pathologic entities of idiopathic interstitial pneumonias, comparing quantitatively the extent of abnormalities in the HRCT and ^99m^Tc-DTPA clearance prospectively. We found that the ^99m^Tc-DTPA clearance time is abnormal in almost all (94.5%) of IPF patients. On the contrary, we failed to detect a strong correlation between HRCT TID score and ^99m^Tc-DTPA clearance. An additional finding of this study is a weak linkage between TID score and eosinophils in BALF.

In a recent prospective study Mogulkoc et al. [16] evaluated the potential ability of ^99m^Tc-DTPA scanning in predicting survival in a large scale population of 106 IPF patients. On the other hand, only 30% of them had histologically proven disease. The authors showed that the predictive value of this technique (in terms of survival) was lower than that of total lung capacity and DLCO. They also detected that the clearance values did not correlate with the HRCT scoreby using a both mono-exponential and bi-exponential analysis [16], in accordance with our data. In line with these findings, a recent published study has shown that clearance of inhaled ^99m^Tc-DTPA is not of value in following the progress of IPF [17]. Mura and coworkers did not detect any significant correlation between ^99m^Tc-DTPA and functional tests or HRCT score [18]. DTPA scan has been used in the repertoire of investigations in a number of diffuse parenchymal diseases [19-25] but all of them were before the recent revision of the histological classification of IPF [26].

The specific mechanism of increased clearance time in IPF is still unclear but has been thought to be secondary to the stretching of epithelial junctions in the alveolar wall as a result of fibrotic traction or lymphocytic infiltration [27,28]. We speculate that the rapid clearance may occur across the bare basement membrane, denuded of epithelium, which has been demonstrated in ultrastructural studies of IPF [29].

We found no correlation between aerosol clearance and pulmonary function parameters, in agreement with the findings of previous studies [16,18-20]. Similarly, only a weak correlation between DTPA clearance and % predicted TLCO was found in patients with sarcoidosis and scleroderma [21-23]. The consistent absence of a correlation may be because the impairment of pulmonary function parameters is a result of the maldistribution of ventilation and perfusion or loss of surface area, while the increased rate of DTPA clearance is caused by increased junctional permeability in the alveolar capillary membranes [16].

Wells et al. reported that depression of TLCO and FVC were positively related to the extent of disease on the CT scan and that there were no independent relationships between functional indices and the pattern or distribution of disease on the CT scan [8,15,30]. We did not find similar correlation between TID score or DTPA and PFTs in our IPF population. We have used TID score in the evaluation of HRCT scans, which is a similar technique of estimating disease extent applied in previous studies [8,11,16,30]. In detail, this method of scoring is similar to that used by Staples and coworkers [12] and Lee and coworkers [13] represents a slight modification of the scoring system used by Wells and coworkers [8,15,30]. It was recently used by our radiologist in the evaluation of dyspnea in IPF [11]. One of the findings of the aforementioned study was that TIDs was significantly associated with FVC, FEV1, TLC and PaO2 [11]. On the other hand, in the studies of Staples [12] and Terriff [31], FVC was not correlated with the HRCT scores. Wells et al. found that the extent of fibrosing alveolitis and the presence of emphysema are independent determinants of functional impairment by using a multivariate analysis model [32]. This observation leads to the premese that quantification of disease severity using pulmonary function tests is often confounded by emphysema [32]. The above discrepancies among previous studies and the present results are difficult to explain but may be related to the variable course of the disease, to the fact that somewhat different methodologies have been used [11,12,15,31,33] and also to the fact that some previous works included patients with interstitial lung disease associated with conditions such as connective tissue disorders [12,15,34]. In addition, we speculate that differences could be derived from the fact that we have not estimated the percentage of emphysema. Furthermore, Kazerooni and coworkers have used a different, in our opinion more complicated, HRCT scoring system [33], and demonstrated an excellent correlation between HRCT and pathology fibrosis score.

We found a moderately significant correlation of DTPA clearance with the visual score in HRCT in 18 patients with IPF. We could not overestimated these results because are based only on a limited number of subjects and on a correlation factor of -0.47; it should be supported by a follow-up study in order to investigate if the clearance of 99m-DTPA can actually predict the clinical course of IPF. Furthermore, a recent study demonstrated that clearance of 99m-DTPA, although abnormal in all subjects in presentation, was not a predictor of disease progression in IPF patients [17]. Additionally, Mogulkoc et coworkers showed that the predictive value of the aforementioned technique (in terms of survival) was lower than that of total lung capacity and DLCO, and the clearance values did not correlate with the HRCT score [17]. However, this study differs from ours in that it focuses on the resolution of the DTPA clearance into fast and slow components. Harrison et al. reported that when the CT scan is normal, abnormalities of BAL and/or DTPA clearance may indicate lung disease at a still earlier stage in patients with systemic sclerosis [24].

We also found a moderate correlation between BAL eosinophilia and HRCT TID score. It has been shown that excess neutrophils and/or eosinophils (>5%) in the BALF have been associated with a higher likelihood of disease progression and a failure to respond to immunosuppression [9].

## Conclusion

Our data suggest that at the moment there are not sufficient evidences to recommend the implementation of ^99m^Tc-DTPA lung scan in the follow-up of IPF patients. Further prospective studies in a large scale and well-defined IPF population are necessary to define the role of this modern technique in the evaluation of pulmonary fibrosis.

## Abbreviations

^99m^Tc-DTPA: inhaled technetium-labeled diethylenetriamine pentaacetate

IPF: Idiopathic Pulmonary Fibrosis

HRCT: High Resolution Computed Tomography

PFTs: pulmonary function tests

BALF: bronchoalveolar lavage fluid

TID: Total Interstitial Disease Score

## Competing interests

The author(s) declare that they have no competing interests.

## Authors' contributions

KMA, NMS and DB participated in the design and coordination of the study. KMA prepared the manuscript. KM carried out the HRCT scoring, NT carried out the Bronchoalveolar Lavage, KP and NK carried out the DTPA scans. NT and EKS carried out the statistical analysis. DB and NMS were invo;ved in revising the article for important intellectual content. All authors read and approved the final manuscript.

## Pre-publication history

The pre-publication history for this paper can be accessed here:



## Figures and Tables

**Table 1:** IPF patients characteristics

Sex (Male/Female)	14/4
Age, range, yr (mean ± SD)	55–81 (67 ± 7.3)
FVC% pred, x ± SD	76.1 ± 11.5
FEV_1_%pred, x ± SD	81.9 ± 14.5
TLC% pred, x ± SD	62.7 ± 9.4
TLCO% pred, x+SD	68.0 ± 12.2

**Table 2:** Mean ± SD of total and percentage (± SD) of differential cell counts in BALF in 18 IPF patients.

Cell Type	
Total cell count, ×10^5^/mL	28.6 ± 3.2
Macrophages,%	82.2 ± 4.3
Lymphocytes,%	7.7 ± 3.0
Neutrophils,%	6.23 ± 2.2
Eosinophils, %	3.7 ± 2.4
